# JAG1-siRNA-Lipid Nanoparticles Suppress Infantile Hemangioma Angiogenesis

**DOI:** 10.3390/pharmaceutics18101322

**Published:** 2026-10-19

**Authors:** Yunfei Teng, Xia Xu, Jie Gao, Jianyong Liu

**Affiliations:** 1 Department of Vascular Surgery, Union Hospital, Tongji Medical College, Huazhong University of Science and Technology, Wuhan 430022, China; tyf224@163.com; 2 Department of Pathology, Union Hospital, Tongji Medical College, Huazhong University of Science and Technology, Wuhan 430022, China; 3 Changhai Clinical Research Unit, The First Affiliated Hospital of Naval Medical University, Shanghai 200433, China

**Keywords:** infantile hemangioma, JAG1, Notch signaling, lipid nanoparticle, siRNA, targeted nanomedicine, gene delivery, angiogenesis

## Abstract

**Background**: Infantile hemangioma (IH) is the most common benign vascular tumor of infancy, and propranolol, the current first-line treatment, requires prolonged twice-daily dosing and has adverse systemic effects. **Methods**: Using the public GSE127487 transcriptomic dataset together with weighted gene coexpression network analysis and protein–protein interaction network analysis, we identified JAG1, a principal endothelial Notch ligand, as the most connected node in the disease protein–protein interaction network, with elevated expression across all clinical stages of IH. To test its use as a drug target, we engineered a lipid nanoparticle (LNP) composed of SM-102/DSPC/cholesterol/DMG-PEG2000 that carried JAG1-specific siRNA. **Results**: At an N/P ratio of 8, the formulation efficiently encapsulated the siRNA, protected it from serum nucleases, and remained colloidally stable. LNP-mediated JAG1 knockdown reduced Matrigel network formation by hemangioma stem cells in vitro. In a hemangioma xenograft model, the intratumoral delivery of JAG1 siRNA reduced tumor volume by approximately 75%, tumor weight by approximately 80%, and microvessel density by more than half, without overt toxicity to the measures collected (body weight and clinical observation). **Conclusions**: In this preclinical model, JAG1 emerges as a credible therapeutic target, and LNP-delivered JAG1 siRNA is a promising gene-silencing strategy against IH angiogenesis.

## 1. Introduction

Infantile hemangioma (IH) is the most common benign vascular tumor of infancy and affects approximately 4–5% of infants. Approximately three to one more girls outnumber boys, and preterm or low-birth-weight infants are at higher risk [1,2,3]. Tumors are built from GLUT1-positive endothelial cells, a marker profile that separates IHs from most other vascular tumors and malformations [4,5]. Its clinical course is also unusually predictable for neoplasms: rapid growth during the first months of life, a plateau, and then slow spontaneous involution over several years [2,3]. Most small lesions can safely be left alone and will involute without sequelae. Hemangiomas near the eye, in the airway, or in the viscera, and those complicated by ulceration, bleeding, or functional impairment are difficult. These need active, sometimes urgent, treatment [3,6].

At the cellular level, IH growth occurs on a dysregulated multipotent hemangioma stem cell (HemSC) compartment that gives rise to immature endothelial cells [7,8]. Lineage tracing and immunophenotyping have shown that the IH-derived endothelium resembles the placental microvasculature, which supports a placental or shared-progenitor origin [7]. The proliferative phase is sustained by an imbalance between pro- and antiangiogenic signals, chief among them vascular endothelial growth factor A (VEGF-A) and the Notch axis [6,8]. Propranolol, the current first-line drug, is thought to work partly by dampening these same programs: it decreases VEGF-A expression and induces endothelial apoptosis [9,10,11].

Propranolol changed the field after the initial report by Léauté-Labrèze and colleagues and the multicenter randomized trial that followed [12,13]. It remains an imperfect drug. Infants take it twice daily for months; hypoglycemia, sleep disturbance, and respiratory infections are recognized as adverse effects, and some hemangiomas respond incompletely or rebound once treatment stops [13,14,15,16]. Topical timolol helps superficial lesions, and various individualized schemes have been explored, each with its own drawbacks [17,18,19]. What is still missing is a treatment that acts on a defined molecular driver locally, without systemic exposure. This gap is the motivation for mechanism-based targeted therapy of the lesion vasculature.

The role of the Notch pathway is obvious. It is a central regulator of vascular development, arterial–venous specification, and endothelial homeostasis [20,21]. Among the canonical ligands, Jagged1 (JAG1) and Delta-like ligand 4 (Dll4) are predominant in the endothelium, and they are activated in opposite directions: Dll4 restrains sprouting through lateral inhibition, whereas Jagged1 antagonizes Dll4 and promotes branching and vascular maturation [22,23,24]. JAG1 dosage clearly matters in humans; haploinsufficiency causes Alagille syndrome, with biliary paucity and congenital cardiovascular anomalies [25]. In IH itself, Boscolo and colleagues reported that Jagged1 signaling steers hemangioma stem cells toward pericyte and vascular smooth muscle differentiation, directly tying JAG1 to vessel maturation in these tumors [26]. What has not existed is a data-driven test of whether JAG1 sits at the center of the IH network plus a concrete way to switch it off.

Silencing a gene in a lesion requires a carrier. Lipid nanoparticles (LNPs) are now the workhorse platform for RNA therapeutics; the COVID-19 mRNA vaccines and the first approved siRNA drug, patisiran, all depend on them [27,28,29]. Our group has worked on hemangioma nanomedicine for years. We previously constructed rapamycin-loaded polymer–lipid hybrid nanoparticles and exosome–mimetic particles-in-microspheres, both of which suppressed hemangioma growth in preclinical models [30,31,32]. In parallel, we developed siRNA delivery systems for cancer, including EGFR-targeted immunoliposomes, anti-VEGFR antibody-conjugated lipid–polymer nanoparticles, and cationic liposomal formulations, applied to hepatocellular carcinoma, gastric cancer, and breast cancer, along with image-guided and localized delivery across several indications [33,34,35,36,37,38,39,40]. This foundation made pointing the same platform at IH a natural step.

In the present study, we analyzed the GSE127487 transcriptome [41] with WGCNA and PPI network analysis, identified JAG1 as the top connected hub gene of the differentially expressed protein–protein interaction network, and tracked the JAG1-Notch axis across clinical stages. We then packaged JAG1 siRNA into an LNP and asked two questions: can it silence JAG1 in HemSCs in vitro, and can it curb growth and angiogenesis in a hemangioma xenograft model in vivo? The answers identify JAG1 as a genuinely tractable target and provide a working nanomedicine for silencing it.

## 2. Materials and Methods

### 2.1. Reagents

Reagents. The ionizable lipids SM-102 (heptadecan-9-yl 8-{(2-hydroxyethyl)[6-oxo-6-(undecyloxy)hexyl]amino}octanoate), DSPC (1,2-distearoyl-sn-glycero-3-phosphocholine), cholesterol, and DMG-PEG2000 (1,2-dimyristoyl-rac-glycero-3-methoxypolyethylene glycol-2000), as well as collagenase, were purchased from MedChemExpress (MCE, Shanghai, China). Blank lipid nanoparticles were prepared using a microfluidic mixing device from XNano (Shanghai, China). JAG1-specific siRNA (sense, 5′-CCUGUAACAUAGCCCGAAAdTdT-3′; antisense, 5′-UUUCGGGCUAUGUUACAGGdTdT-3′) and scrambled negative control siRNA (sense, 5′-UUCUCCGAACGUGUCACGUdTdT-3′; antisense, 5′-ACGUGACACGUUCGGAGAAdTdT-3′) were synthesized by Shanghai BiOligo Biotech Co., Ltd. (Shanghai, China); all the strands carried 3′-terminal dTdT overhangs, with no additional chemical modifications, and were purified by HPLC. CD133 magnetic beads were obtained from Miltenyi Biotec (Bergisch Gladbach, Germany), Matrigel from Corning (Bedford, MA, USA), and EGM-2 medium from Lonza (Basel, Switzerland).

### 2.2. Bioinformatic Analysis

Data acquisition and preprocessing. The GSE127487 dataset was downloaded from the Gene Expression Omnibus (GEO) using the GEOquery package [42]. This dataset was generated on the Illumina HumanHT-12 V4.0 BeadChip (platform GPL10558) and comprises 28 samples: 5 normal skin controls, 18 IH specimens spanning three clinical stages (6-month proliferative, *n* = 6; 12-month plateau (stable), *n* = 6; 24-month involuting, *n* = 6), and 5 propranolol-treated samples [41]. Five propranolol-treated samples were excluded to focus on the untreated natural history of IH, leaving 23 samples (18 IH, 5 normal) for downstream analysis. GSE127487 provides no covariates beyond stage (its only sample annotations are tissue, age at sampling, and propranolol status, with age collinear with stage in the untreated cohort); thus, no covariate adjustment was performed. Raw signal intensities were log2-transformed and quantile-normalized using the normalizeQuantiles function in limma [43], a step that removes technical intensity variation across arrays while preserving biological signals. Probe identifiers were mapped to official gene symbols; where multiple probes mapped to the same gene, the probe with the highest mean expression was retained as the representative measurement, yielding a final matrix of 27,538 unique genes. All bioinformatic analyses were performed in the R statistical environment (version 4.x) with Bioconductor packages.

Differential expression analysis. Differential expression between IH (*n* = 18) and normal skin (*n* = 5) was assessed using limma [43] with empirical Bayes moderated t tests, which borrow information across genes to stabilize variance estimates and are well suited for small-sample microarray studies. Differentially expressed genes (DEGs) were defined by |logFC| > 1 and Benjamini–Hochberg adjusted *p* < 0.05, yielding 1966 DEGs (1753 upregulated and 213 downregulated).

WGCNA. Weighted gene coexpression network analysis was performed using the WGCNA package [44] on all 27,538 genes. A soft-thresholding power of β = 12 was selected on the basis of the scale-free topology fit (SFT. R^2^ = 0.965, slope = −1.54, mean connectivity = 25.1), where a higher β emphasizes strong correlations and produces a more stringent network. An unsigned topological overlap matrix (TOM) was constructed to capture the shared-neighbor structure of the coexpression network, and hierarchical clustering with dynamic tree cutting (minimum module size = 100, merge cut height = 0.15) was used to define the gene modules. Module eigengenes were correlated with IH status by Pearson correlation. The ME3 module (1531 genes; r = 0.882; *p* = 2.65 × 10^−8^) exhibited the strongest correlation with IH, and its 822-gene overlap with the DEG list was carried forward for downstream analysis. The choice of β was robust: at β = 8 and β = 14 (same unsigned TOM, minimum module size, and merge cut height), the top trait-correlated module remained ME3 (r = 0.874 and 0.883; membership Jaccard vs. β = 12: 0.553 and 0.747), and bootstrap resampling (1000 resamples) gave r = 0.882 (95% CI 0.729–0.950).

Functional enrichment analysis. Overrepresentation analysis (ORA) of Gene Ontology (GO) and Kyoto Encyclopedia of Genes and Genomes (KEGG) terms among the 822 overlapping genes was performed using clusterProfiler [45], with *p* values adjusted for multiple testing using the Benjamini–Hochberg procedure. Gene set enrichment analysis (GSEA) [46] was applied to all 27,538 genes ranked by logFC using the fgsea package (v1.32.0; Korotkevich et al., bioRxiv 2021) with 10,000 permutations (set.seed = 42) to identify gene sets with coordinated, threshold-free enrichment. Single-sample GSEA (ssGSEA) was performed using the GSVA package [47] with the “ssgsea” method, adapted from the approach of Barbie et al. [48], to estimate sample-level pathway activity for the custom gene sets PROLIFERATION, NOTCH, ECM_ORGANIZATION, ANGIOGENESIS, and ME3 (gene-set definitions provided in Table S1). Differences across clinical stages were assessed by one-way ANOVA.

PPI network and hub genes. Protein–protein interaction (PPI) analysis of the 822 overlapping genes was performed using STRING v12.0 with a confidence score threshold of 0.4 [49], after which interactions were filtered to medium or high confidence. Degree centrality, defined as the number of direct interaction partners, was used to rank and identify hub genes. Genes without any interaction partners in STRING (i.e., isolated nodes) were excluded, yielding a 498-node network for visualization and hub-gene analysis. The same STRING procedure was additionally applied to the full 1966-gene DEG list, yielding an 828-node network used for the JAG1-centered Notch-cluster analysis (Figure S4). Community detection was performed using the cluster_louvain algorithm implemented in the igraph package to resolve functionally coherent submodules within the network.

### 2.3. LNP Preparation and Characterization

Preparation of blank LNPs. The ionizable lipids SM-102, DSPC, cholesterol, and DMG-PEG2000 were dissolved in anhydrous ethanol at a molar ratio of 50:10:38.5:1.5 to form the organic phase; the aqueous phase consisted of sodium acetate buffer (pH 4.0), which protonated the ionizable lipid to facilitate subsequent siRNA loading. The two phases were mixed at a 1:3 (organic:aqueous) volume ratio with a total flow rate of 2 mL/min using a microfluidic mixing device, triggering rapid lipid self-assembly into nanoparticles. The residual ethanol was removed, and the buffer was exchanged by ultrafiltration through a 100 kDa MWCO membrane (Millipore, Merck, Darmstadt, Germany) into PBS (pH 7.4) to yield a neutral-pH nanoparticle dispersion. The resulting LNPs were collected by centrifugation at 3000× *g* at 4 °C, stored at 4 °C and used within 7 days of preparation.

siRNA loading and N/P optimization. JAG1 siRNA was loaded postinsertion: siRNA was added to the blank LNPs at nitrogen-to-phosphate (N/P) ratios of 4, 6, 8, and 10, where the N/P ratio is defined as the molar ratio of ionizable-lipid amine nitrogen to the siRNA backbone phosphate. The mixture was incubated at 37 °C for 30 min to allow electrostatic association and encapsulation of the negatively charged siRNA by the positively charged ionizable lipid, a loading strategy that avoids exposing the siRNA to the organic phase during particle formation.

Size and zeta potential. Samples (50 μL diluted to 1 mL in PBS, pH 7.4) were passed through a 0.22 µm filter and analyzed by dynamic light scattering (DLS; Malvern Zetasizer) for mean particle size (Z-average), polydispersity index (PDI), and zeta potential. DLS measures the hydrodynamic diameter and size distribution of the nanoparticles, and the PDI reflects the width of that distribution; the zeta potential, determined by electrophoretic mobility, reflects the surface charge that governs colloidal stability.

siRNA encapsulation efficiency. Agarose gel electrophoresis was used to compare free versus encapsulated siRNA across the different N/P ratios. Encapsulated siRNA was released by treatment with 10% Triton X-100 to evaluate total loading. Quantitative loading was measured by the RiboGreen assay: siRNA was quantified fluorometrically (excitation 485 nm/emission 520 nm) against a standard curve (0–1000 ng/mL) without (free siRNA) and with (total siRNA) 0.5% Triton X-100 permeabilization; loading efficiency was calculated as (total − free)/total × 100 across three independent batches.

Stability. siRNA-LNPs (N/P = 8) were stored at 4 °C, and their size was measured on days 1, 3, 5, 7 and 14 to assess their storage stability. For serum stability, N/P = 8 LNPs were incubated with fetal bovine serum (FBS) (50% volume/volume) at 37 °C for 0, 6 and 24 h, with free siRNA as a control; following Triton X-100 treatment to release encapsulated siRNA, the samples were resolved by agarose gel electrophoresis to evaluate the protection of the siRNA from nuclease degradation in serum. The serum stability comparison is based on this single archived gel, scored semiquantitatively by band presence and relative intensity; uneven background illumination across the plate precludes reliable quantitative lane densitometry.

### 2.4. Cell Culture and In Vitro Functional Assays

Transfection. For knockdown experiments, HemSCs were seeded and allowed to adhere before transfection using our laboratory-developed LNP carrier at a final siRNA concentration of 100 nM. Transfected cells were subsequently analyzed by qRT–PCR, Western blotting, and Matrigel network formation assays. For uptake analysis, FITC-conjugated JAG1 siRNA was loaded into blank LNPs by the same postinsertion procedure at N/P 8; HemSCs were incubated with FITC-siRNA-LNPs at 100 nM siRNA equivalent, counterstained with DAPI, and analyzed by flow cytometry in the FITC channel, with free FITC-siRNA as the control.

Cell culture. This study was approved by the Research Ethics Committee of Wuhan Union Hospital (Wuhan, China) (Approval No. 09865), and written informed consent was obtained from all patients. In accordance with ethical and legal standards, all human specimens were processed anonymously, and the diagnosis was confirmed by the Department of Pathology of Wuhan Union Hospital. Following excision, hemangioma-derived stem cells (HemSCs) were isolated and cultured using our previously described procedures [30]. Briefly, infantile hemangioma tissue was digested with collagenase (0.2 mg/mL) at 37 °C, after which the tissue was dissociated into single cells. The resulting single-cell suspension was washed with phosphate-buffered saline (PBS) and centrifuged to obtain a cell pellet. After resuspension, CD133+ HemSCs were isolated using a CD133 magnetic bead isolation kit according to the manufacturer’s instructions. The purity of the selected CD133+ population was 93%, as determined by flow cytometry on the day of sorting. All the experiments used this single-patient isolate; specimens from different donors were not pooled. The isolated HemSCs were cultured in endothelial growth medium-2 (EGM-2) supplemented with 20% fetal bovine serum (FBS).

Quantitative real-time PCR (qRT–PCR). Total RNA was extracted using RNA Isolater reagent (Vazyme R401-01) and reverse-transcribed into cDNA using HiScript II Q RT SuperMix (Vazyme R223-01). qRT–PCR was performed using ChamQ SYBR qPCR Master Mix (Vazyme Q311-01) on a real-time PCR system. Relative JAG1 expression was calculated using the 2^−ΔΔCT^ method, with 18S ribosomal RNA used as the internal reference to normalize the amount of input RNA. The sequences of the primers used were as follows: JAG1 forward, 5′-AATGGCTACCGGTGTGTCTG-3′ and reverse, 5′-CCCATGGTGATGCAAGGTCT-3′; 18S forward, 5′-CGACGACCCATTCGAACGTCT-3′ and reverse, 5′-CTCTCCGGAATCGAACCCTGA-3′.

Western blotting. Cells were lysed in radioimmunoprecipitation assay (RIPA) buffer containing phenylmethanesulfonyl fluoride (PMSF) and protease inhibitors, and the total protein concentration was quantified using a bicinchoninic acid (BCA) assay. Equal amounts of protein were resolved by sodium dodecyl sulfate–polyacrylamide gel electrophoresis (SDS–PAGE) and transferred to polyvinylidene difluoride (PVDF) membranes. The membranes were blocked in nonfat milk to reduce nonspecific binding and probed with anti-JAG1 (Proteintech 66890-1-Ig, 1:500) and anti-Tubulin (Proteintech 10094-1-AP, 1:1000) primary antibodies overnight at 4 °C. After being washed, the membranes were incubated with horseradish peroxidase (HRP)-conjugated secondary antibodies (anti-rabbit SA00001-2, 1:10,000; anti-mouse SA00001-1, 1:10,000) for 1 h at room temperature, after which the protein bands were visualized by enhanced chemiluminescence (ECL). Tubulin served as the loading control, and band intensities were quantified by densitometry for normalization.

Network formation assay (Matrigel). Matrigel (50 μL/well) was polymerized at 37 °C for 45 min to form a basement membrane substrate. Transfected HemSCs were seeded at 3 × 10^4^ cells/well in six replicate wells and incubated for 4–6 h at 37 °C with 5% CO_2_ in growth factor-supplemented medium until stable networks formed. The total segment length of the networks formed by HemSCs, a readout of the Matrigel network-forming phenotype, was quantified from phase-contrast images using ImageJ software (version 1.54p, NIH, Bethesda, MD, USA).

### 2.5. In Vivo Antitumor Study

Female BALB/c nude mice (6–8 weeks of age) were purchased from the Experimental Animal Center of Tongji Medical College (Wuhan, China). All animal procedures were approved by the Research Ethics Committee of Wuhan Union Hospital (No. 00036) and performed in accordance with institutional guidelines.

**Figure 6:**
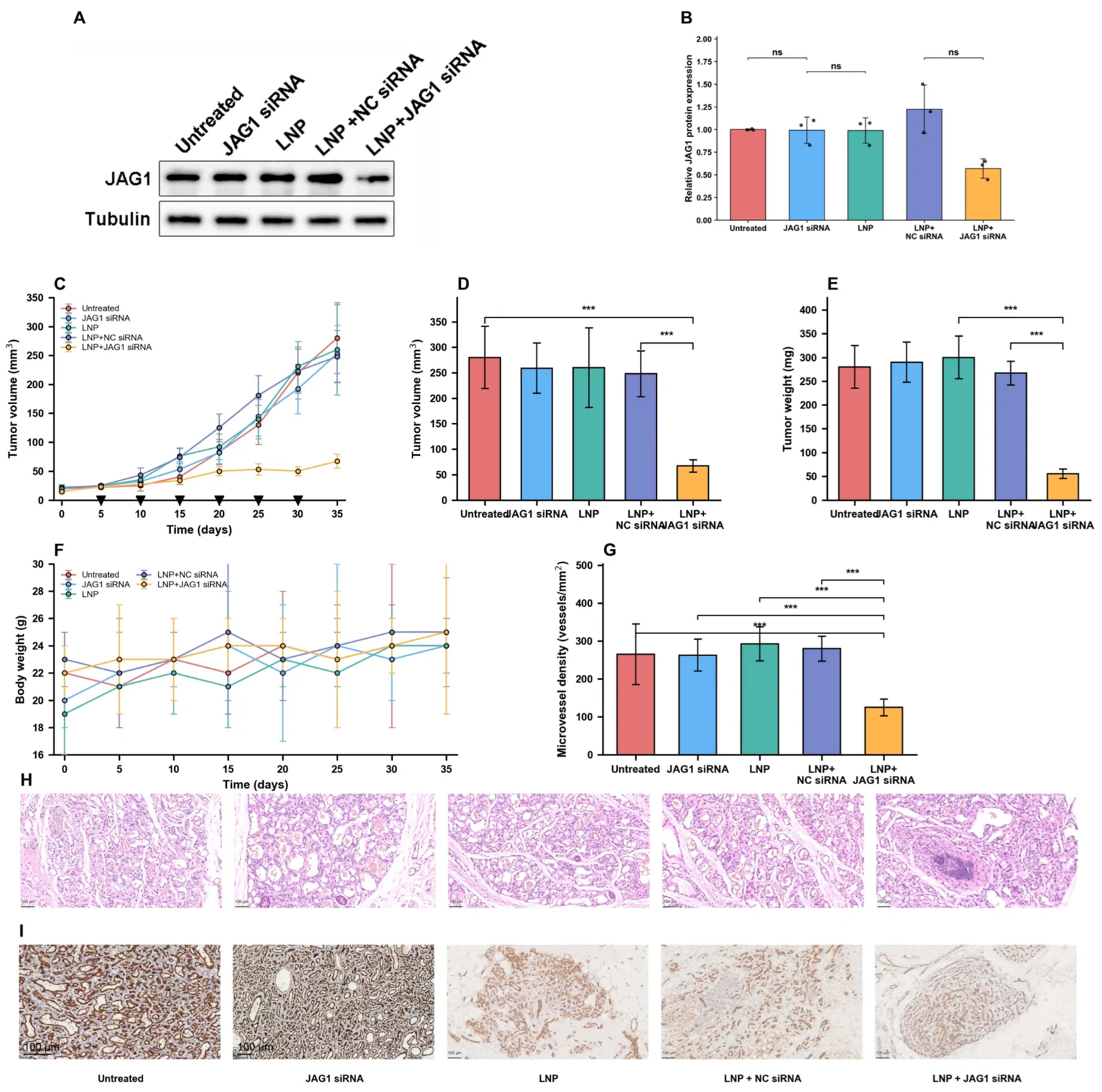
In vivo antitumor efficacy. (**A**,**B**) Western blot and densitometric quantification of JAG1 in excised tumors normalized to tubulin. The data are presented as the mean ± SD (n = 3). (**C**) Tumor growth curves over time; downward triangles mark the intratumoral dosing days (5, 10, 15, 20, 25, and 30) (trend display; mean ± SD, n = 6; shown without per-time-point significance marks because per-animal longitudinal records were not archived). (**D**) Final tumor volume at the day 35 endpoint. (**E**) Tumor weight at the endpoint. (**F**) Body weight across groups, indicating no overt systemic toxicity. (**G**) Microvessel density quantified on H&E-stained sections as vessels containing red blood cells per mm^2^ (mean ± SEM; *** *p* < 0.001 versus LNP+JAG1 siRNA; one-way ANOVA with the Newman–Keuls post hoc test). (**H**) Representative H&E staining of excised hemangiomas showing the lesional vascular architecture (original magnification, 200×). (**I**) Representative CD31 immunohistochemistry results for each group (20× scans; 100 µm scale bars are shown on the Untreated and JAG1 siRNA scans; the remaining scans are exported without scale bars). For (**D**,**E**), the data are presented as the mean ± SD (n = 6), and the significance values refer to the day 35 endpoint comparisons (**D**,**E**). *** *p* < 0.001 versus LNP+JAG1 siRNA. ns, not significant.

Hemangioma xenografts were established by suspending HemSCs at 1 × 10^7^ cells in Matrigel and inoculating them subcutaneously into the flank of each mouse. The mice were randomized into five groups (untreated, JAG1 siRNA, LNP, LNP+NC siRNA, and LNP+JAG1 siRNA) when the tumor volume reached approximately 25 mm^3^ on day 0, after which they were treated by intratumoral injection at a siRNA dose of 5 mg/kg (100 µL per injection), a local RNA–LNP dose with a xenograft precedent [50], and on days 5, 10, 15, 20, 25, and 30; the therapeutic payload was delivered directly to the lesion via the intratumoral route. The negative control (NC) siRNA denotes a nontargeting scrambled siRNA of equal length and chemical composition. Tumor volume and body weight were measured over time; at the experimental endpoint, tumors were excised, weighed, fixed in paraformaldehyde, and paraffin-embedded for sectioning. For microvessel density (MVD) analysis, H&E-stained sections were used; microvessels were quantified by counting lumens containing red blood cells, and the MVD was expressed as vessels/mm^2^. Tumor measurements and histological quantification were performed by blinded operators. New CD31 and GLUT1 immunohistochemistry was additionally performed on archived paraffin sections of the excised xenografts (one archived section per group for CD31; all sections additionally available for new staining were stained for GLUT1 on separate sections). The primary antibodies used were rat anti-mouse CD31 monoclonal antibody (clone SZ31; Dianova, DIA-310; 1:100) and rabbit anti-GLUT1 polyclonal antibody (Proteintech, 21829-1-AP; 1:500); the mouse-specific CD31 reagent selectively detected host-derived murine endothelium in the xenografts. Sections were scanned at 20× original magnification, and representative fields are shown (CD31, Figure 6I; GLUT1, Figure S8). Because only one archived section per group was available for the new staining, and field-level measurements within a single section cannot separate between-section variability from treatment effects, the new CD31 staining is presented as a qualitative morphological confirmation of the endothelial compartment; quantitative vascular inference is anchored to the H&E-based microvessel density, which was quantified at the animal level by blinded operators.

In a separate cohort for in vivo silencing assessment, tumor-bearing mice received a single intratumoral injection of LNP+JAG1 siRNA at a dose of 5 mg/kg, with LNP+NC siRNA, LNP alone, and untreated mice serving as controls. At 48 h postinjection, the tumors were excised and homogenized, and JAG1 protein levels were determined by standard Western blotting with densitometric quantification and normalized to tubulin levels. The relative JAG1 protein expression in each group was calculated and compared with that in the untreated control group.

### 2.6. Statistical Analysis

Statistical analysis. Data are presented as the mean ± SD as indicated. Comparisons between two groups were performed using Student’s two-tailed t test, and comparisons among three or more groups were performed using one-way analysis of variance (ANOVA) followed by the Newman–Keuls post hoc test, which is consistent with our prior hemangioma studies. The Newman–Keuls procedure controls the familywise error rate across multiple pairwise comparisons, making it appropriate for the multigroup designs used here. Statistical significance was defined as *p* < 0.05 (*), *p* < 0.01 (**), and *p* < 0.001 (***). The JAG1 qPCR time course was analyzed by two-way ANOVA (treatment × time) on replicate-level ΔCt values. For the in vivo study, per-animal longitudinal records were not archived; growth curves are therefore presented as trend displays, and statistical inference is anchored to the day-35 endpoint readouts (tumor volume, tumor weight, and MVD). All the statistical analyses were performed using GraphPad Prism 9 (GraphPad Software, San Diego, CA, USA).

## 3. Results

### 3.1. Transcriptomic Landscape and Functional Enrichment of Infantile Hemangioma

**Figure 1:**
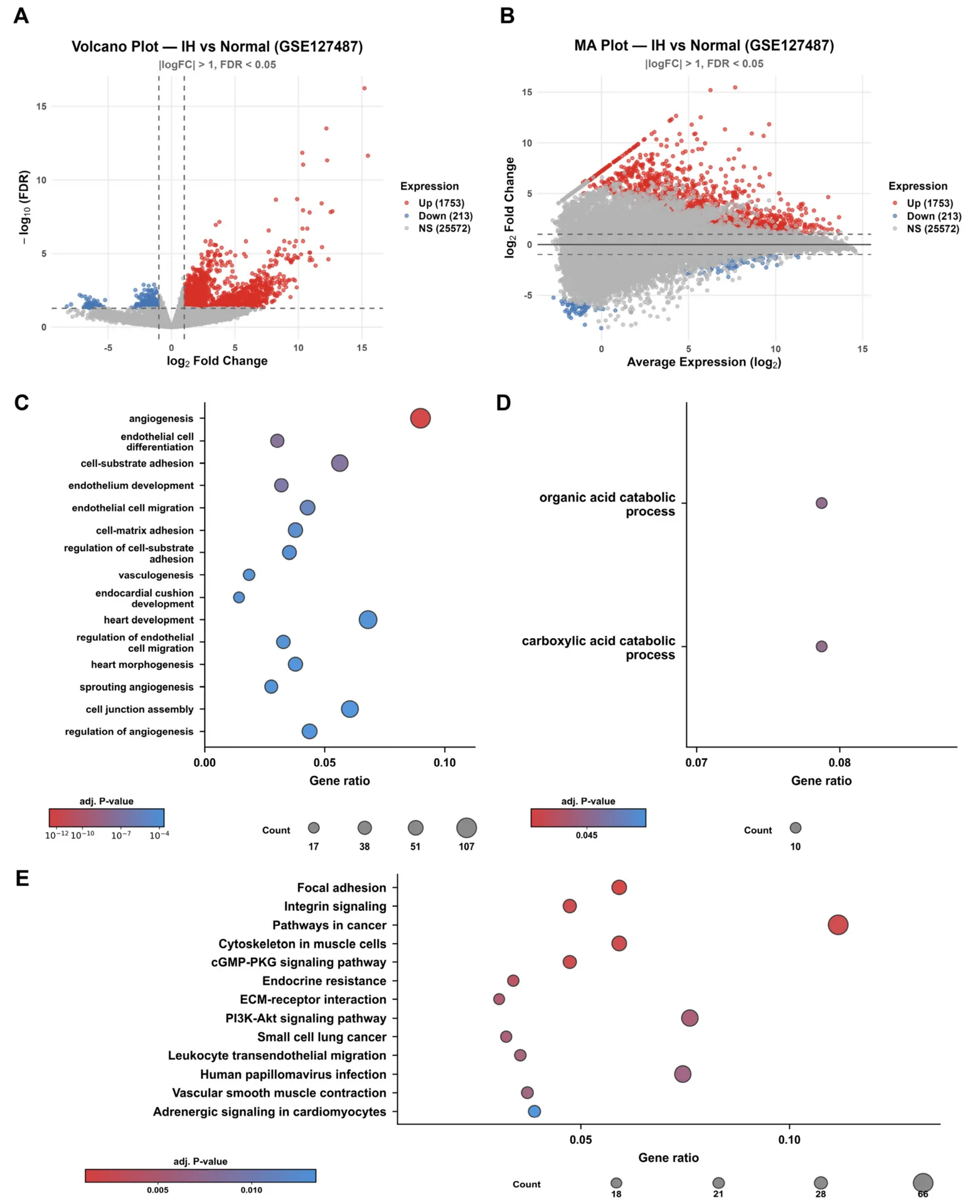
Transcriptomic landscape of infantile hemangioma. (**A**) Volcano plot of differentially expressed genes (DEGs) between IH (n = 18) and normal skin (n = 5). Red dots denote upregulated genes (1753), and blue dots denote downregulated genes (213), defined at |logFC| > 1 and adjusted *p* < 0.05 (Benjamini–Hochberg). (**B**) MA plot showing the log fold change against mean expression, illustrating that differential expression spanned the entire abundance range. (**C**) Top Gene Ontology (GO) biological process terms overrepresented among upregulated DEGs, ranked by adjusted *p* value. (**D**) GO terms enriched among the downregulated DEGs, namely, organic acid catabolic process and carboxylic acid catabolic process. (**E**) The top KEGG pathways identified by overrepresentation analysis.

We first compared IH tissue (n = 18) with normal skin (n = 5) in the GSE127487 dataset. At thresholds of |logFC| > 1 and Benjamini–Hochberg adjusted *p* < 0.05, 1966 genes were differentially expressed: 1753 upregulated genes and 213 downregulated genes (Figure 1A). An up-to-down ratio of 8.2:1 indicates something simple about the lesion. Its transcriptional program is predominantly activated and proliferative, in line with the endothelial hyperproliferation of the growth phase. Because the IH group spans the proliferative, plateau, and involution stages, these DEGs capture features shared across the disease rather than at a single time point. The MA plot (Figure 1B) revealed that the differences were not confined to any expression range but spanned the whole abundance spectrum. Among the genes whose expression was most strongly induced were LIN28B (logFC = 15.21), TOP2A (logFC = 4.72), and NOTCH4 (logFC = 4.58), which are related to pluripotency, proliferation, and Notch signaling, respectively. The full per-stage differential expression results are provided in Table S2.

Which pathways do these genes populate? GO analysis of the 1753 upregulated DEGs revealed “angiogenesis” as the top term (107 of 1190 genes; adjusted *p* = 2.67 × 10^−13^), followed by “endothelial cell differentiation” and “cell–substrate adhesion” (Figure 1C); overrepresentation analysis of the 822 genes shared between the ME3 module and the DEG list likewise ranked cell-substrate adhesion, endothelial cell migration, and regulation of angiogenesis among its top terms. The downregulated genes clustered instead in catabolism, namely, “organic acid catabolic process” and “carboxylic acid catabolic process” (Figure 1D), suggesting that the core metabolic programs are relatively suppressed inside the lesion. KEGG analysis of the upregulated genes highlighted “Focal adhesion” and “Integrin signaling”, followed by “ECM–receptor interaction” and “PI3K–Akt signaling pathway” (Figure 1E). Threshold-free GSEA over all 27,538 genes ranked by logFC gave the highest enrichment score to “vascular endothelial cell proliferation” (NES = 2.024, *p* < 0.001), and KEGG-based GSEA flagged the “cell cycle” pathway (Figure S1). One picture emerges: a proliferative endothelial signature is predominant in IH.

### 3.2. WGCNA Identifies the ME3 Module and a JAG1-Notch Hub

**Figure 2:**
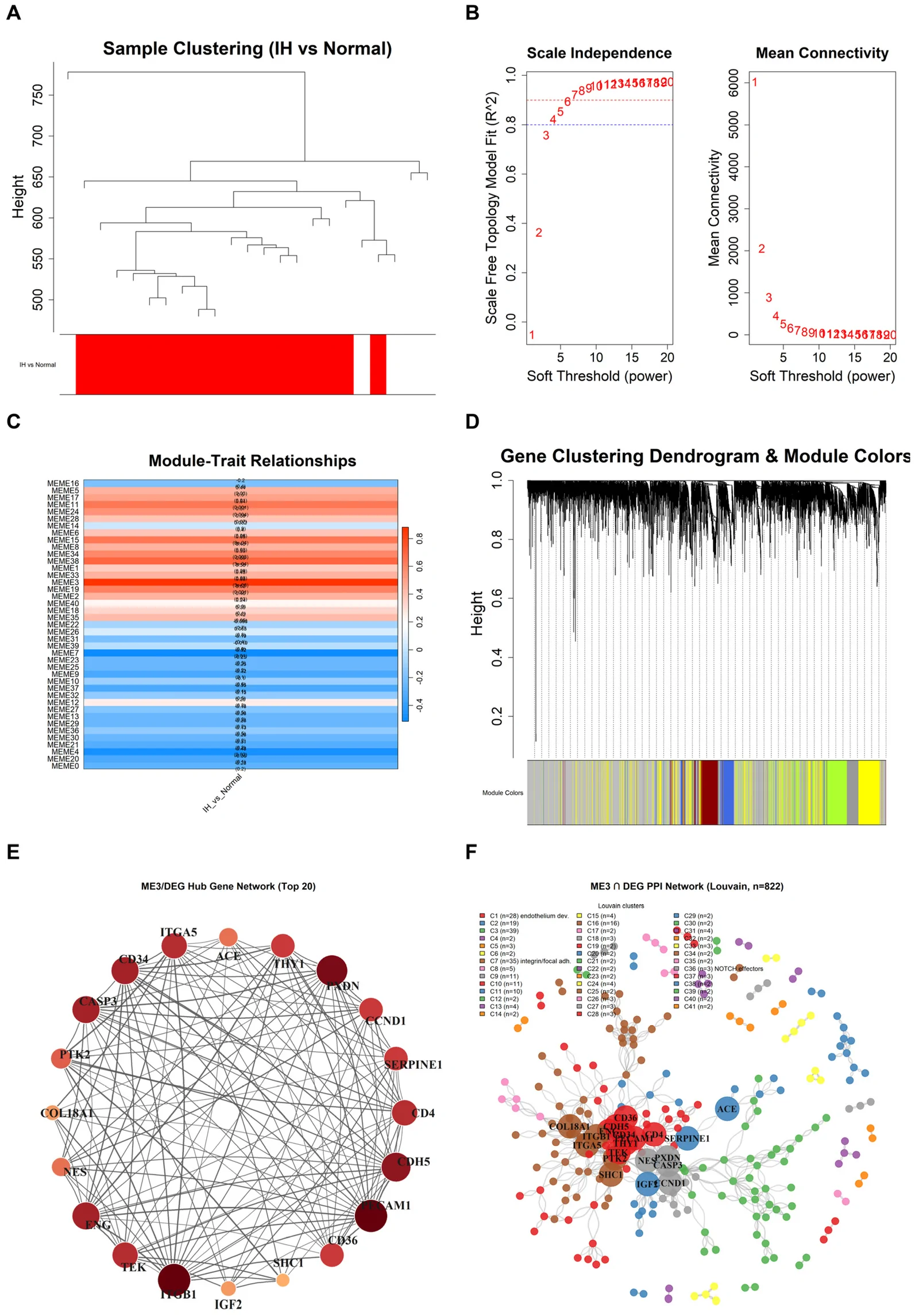
WGCNA and PPI network analysis revealed JAG1 as a hub gene. (**A**) Sample dendrogram and trait heatmap showing no outlier samples. (**B**) Selection of the soft-thresholding power (β = 12) on the basis of scale-free topology fit and mean connectivity. (**C**) Module–trait correlation; the ME3 module has the strongest correlation with IH status (r = 0.882, *p* = 2.65 × 10^−8^). (**D**) Hierarchical clustering dendrogram of gene modules. (**E**) Circular protein–protein interaction (PPI) network of the top 20 ME3/DEG hub genes; node size reflects degree centrality. (**F**) Full PPI network of the ME3/DEG overlap with Louvain community detection, resolving functionally distinct clusters, including an endothelium development cluster (C1), an integrin/focal adhesion cluster (C7), and a small cluster of Notch transcriptional effectors (C36; JAG2, HEY1, and HEYL).

WGCNA was applied to all 27,538 genes. Sample clustering revealed no outliers (Figure 2A). We chose a soft-thresholding power of β = 12 and constructed an unsigned network (Figure 2B); hierarchical clustering with dynamic tree cutting separated the transcriptome into modules (Figure 2D). Among these modules, the ME3 module tracked the IH status most closely (1531 genes; Figure 2C). We took the 822 genes that ME3 shares with the DEG list into STRING and ranked the resulting network by degree. The top five hubs were CDH5, ITGB1, CD4, PECAM1, and CD34—a compact endothelial identity core (Figure 2E). Louvain community detection then split the network into coherent subgroups, including an endothelium development cluster (Cluster 1, top GO term), an integrin/focal adhesion cluster (Cluster 7), and a small cluster of Notch transcriptional effectors (JAG2, HEY1, and HEYL; Cluster 36) (Figure 2F). All 20 hub genes were expressed at higher levels in IH tissue than in normal tissue (Figure S2). Within the top cluster, the endothelial identity genes PECAM1, CDH5, CD34, TEK, and ENG increased together (Figure S3); when the PPI was built from the full DEG list, JAG1 emerged as the most connected node overall and anchored a Notch signaling cluster containing JAG2, NOTCH3, NOTCH4, HEY1, HEYL, MFNG, ADAM17, PSEN1, and LFNG (Figure S4). A strong proliferative DEG signature therefore coexists with vascular identity and Notch programs in the IH transcriptome.

### 3.3. JAG1 Is Robustly Upregulated Across Stages with a Persistent Notch Gene-Set Signature

**Figure 3:**
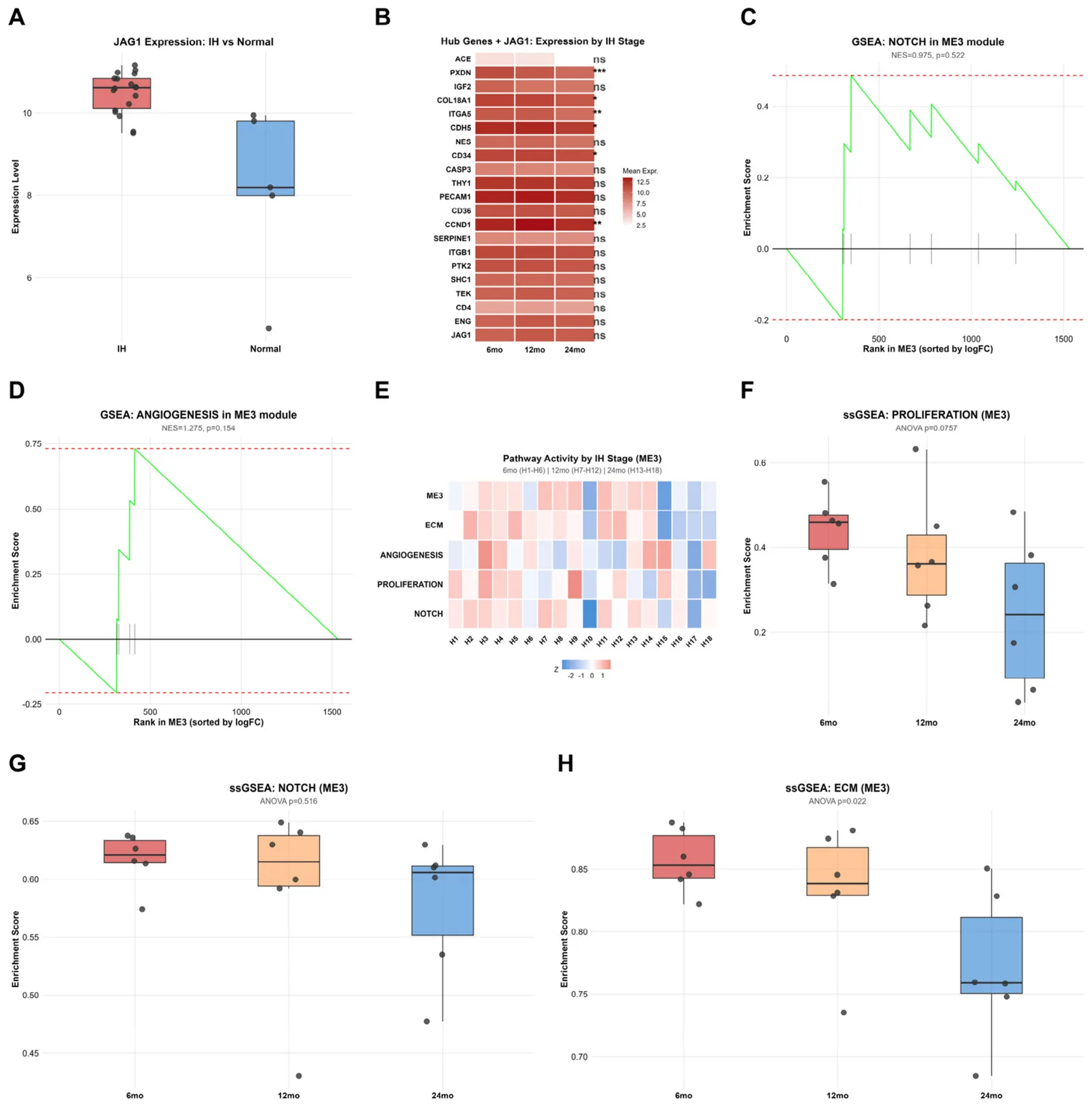
JAG1 expression and pathway dynamics across IH stages. (**A**) Box plot of JAG1 expression in IH versus normal tissue (logFC = 2.34, adjusted *p* = 0.006; limma moderated t test with Benjamini–Hochberg correction). (**B**) Stagewise expression of the corrected ME3/DEG hub genes and JAG1 across the 6-, 12-, and 24-month clinical stages; per-gene one-way ANOVA with the Newman–Keuls post hoc test (* *p* < 0.05, ** *p* < 0.01, *** *p* < 0.001; ns, not significant). (**C**,**D**) Gene set enrichment analysis within the corrected ME3 module for Notch signaling (NES = 0.975; *p* = 0.52) and angiogenesis (NES = 1.275; *p* = 0.15); neither reached significance. (**E**) Single-sample GSEA (ssGSEA) heatmaps of pathway activity across stages. (**F**–**H**) ssGSEA scores for proliferation, Notch signaling, and extracellular matrix (ECM) organization across stages; box plots show the median, interquartile range, and range.

JAG1 itself stood out for how uniformly it was elevated. The mean expression was 10.485 in IH versus 8.145 in normal skin (logFC = 2.34, *p* = 0.006; Figure 3A), with a coefficient of variation across IH specimens of only 4.74%. Stagewise profiling revealed a more interesting story (Figure 3B; stage-annotated sample distribution in Figure S5). The expression of several hub genes decreased with involution: the expression of PXDN, ITGA5, and COL18A1 decreased monotonically and significantly toward the 24-month stage (one-way ANOVA, *p* = 2.7 × 10^−5^, 3.0 × 10^−3^, and 0.011), whereas that of the endothelial identity hubs PECAM1, TEK, and ENG remained nearly constant. JAG1 did not follow the declining genes either. It was numerically highest at 12 months (10.23 at 6 months, 10.76 at 12 months, and 10.47 at 24 months), but the differences were not significant (one-way ANOVA, *p* = 0.18); thus, we treat this only as a nonsignificant trend. Compared with normal skin, however, JAG1 remained elevated at every stage (log2FC 2.08, 2.62, and 2.32 at 6, 12, and 24 months; nominal *p* = 0.033, 0.010, and 0.020, respectively; per-stage BH-adjusted P nonsignificant given the limited sample sizes; pooled BH-adjusted *p* = 0.006); thus, the ANOVA result is read as the absence of a stage trend rather than as evidence against sustained elevation. In the corrected ME3 module, curated-set GSEA revealed no significant enrichment for the Notch (NES = 0.975; *p* = 0.52; Figure 3C) or angiogenesis (NES = 1.275; *p* = 0.15; Figure 3D) sets, and the two curated sets were testable within the module (the proliferation set was absent from the module, and the ECM set fell below its minimum membership; full-size enrichment curves in Figure S6), indicating that the proliferation and Notch signals are carried at the DEG and DEG-PPI layers rather than within the module itself. Sample-level ssGSEA heatmaps were used to summarize the pathway activity for the ME3, ECM organization, angiogenesis, proliferation, and Notch gene sets across stages (Figure 3E). The proliferation scores decreased across stages (0.441, 0.381, and 0.241 at 6, 12, and 24 months; *p* = 0.076; Figure 3F), the Notch scores remained steady (*p* = 0.52; Figure 3G), and the degree of ECM organization decreased significantly (*p* = 0.022; Figure 3H). In short, the expression of proliferation markers fades as the lesion becomes involuted, but JAG1 expression and the Notch gene-set signature persist. That persistence keeps JAG1 available as a target throughout the disease course.

### 3.4. siRNA-LNP Characterization and Optimization

**Figure 4:**
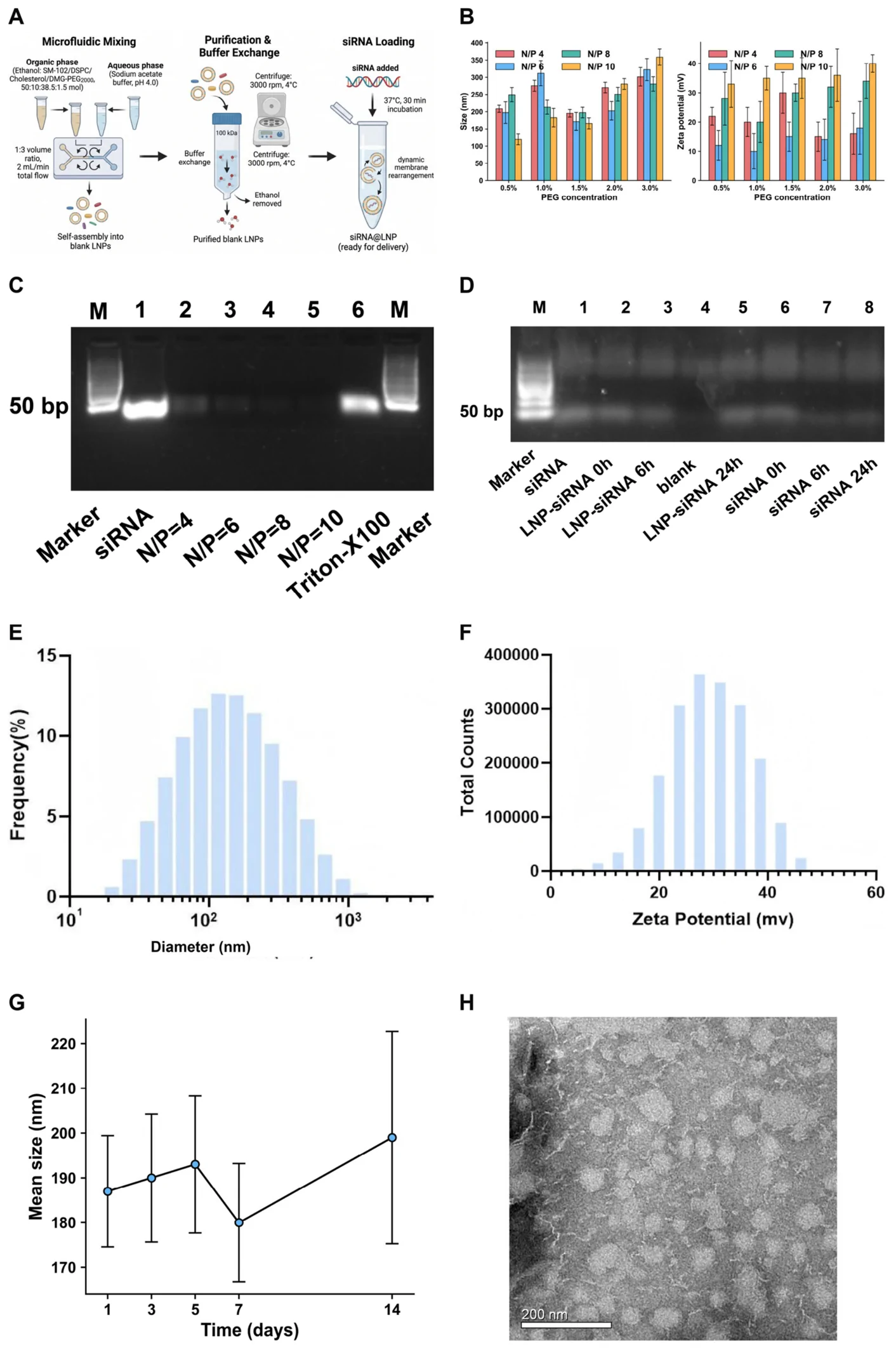
siRNA-LNP formulation and characterization. (**A**) Schematic of the microfluidic preparation of blank LNPs followed by postinsertion loading of JAG1 siRNA. The figure was created by Figdraw (www.figdraw.com). (**B**) Particle size and zeta potential of siRNA-LNPs across N/P ratios of 4, 6, 8, and 10 (n = 3 per group). (**C**) Agarose gel electrophoresis showing siRNA encapsulation across N/P ratios; free siRNA was undetectable at all ratios tested (4–10) and was released by Triton X-100 treatment. (**D**) Serum stability of N/P = 8 LNPs compared with that of free siRNA over 24 h, as demonstrated by protection from nuclease degradation. (**E**) Size distribution and (**F**) zeta potential of the selected N/P = 8 formulation. (**G**) Storage stability of siRNA-LNPs at 4 °C over 14 days, showing that the N/P = 8 formulation maintained its colloidal stability throughout the storage period. (**H**) Negatively stained transmission electron micrograph of the final N/P = 8 formulation (scale bar, 200 nm), documenting particle morphology; all size and polydispersity quantification is by DLS (**B**,**E**), and dry-state negative-stain dimensions are expected to be smaller than the DLS hydrodynamic diameters. The data in (**B**,**D**,**E**–**G**) are the mean ± SD (n = 3).

We prepared blank LNPs from SM-102/DSPC/cholesterol/DMG-PEG2000 (50:10:38.5:1.5, molar) by microfluidic mixing and loaded JAG1 siRNA by postinsertion (Figure 4A). To identify a working formulation, we varied the N/P ratio (4, 6, 8, and 10) and the PEG content and measured the particle size and zeta potential (Figure 4B). With respect to the selected 1.5% DMG-PEG2000, the mean sizes were 195.5 ± 10.9, 171.6 ± 26.3, 197.6 ± 15.3, and 166.4 ± 15.9 nm at N/P 4, 6, 8, and 10, respectively, with zeta potentials of +30 ± 7, +15 ± 5, +30 ± 3, and +35 ± 7 mV, respectively. The zeta potential tended to increase with increasing N/P ratio—N/P 6 (+15 ± 5 mV) was the exception—which was consistent with more protonated amines becoming available for charge pairing at higher amine excess, while increasing the PEG content enlarged the particles by thickening the hydration shell. We selected 1.5% DMG-PEG2000 as the best compromise among size, surface charge, and downstream colloidal stability. On agarose gels, free siRNA was no longer detectable at any N/P ratio tested (4–10) and reappeared only after Triton X-100 disruption (Figure 4C). RiboGreen quantification of the working formulation revealed a loading efficiency of 90.0 ± 1.2% (mean ± SD, three independent batches), with a total associated siRNA concentration of 81.4 ± 1.4 µg/mL. Encapsulation was therefore already complete at the lowest ratio, and a working N/P of 8 was chosen on size, charge, and stability grounds. N/P 6 resulted in the lowest surface charge (+15 ± 5 mV), and N/P 10 resulted in the highest surface charge (+35 ± 7 mV, together with the largest lipid-to-siRNA ratio), whereas N/P 8 combined with a mid-range, batch-reproducible positive charge (+30 ± 3 mV) with complete loading; N/P 4 was nearly indistinguishable from N/P 8 in terms of size and charge, and a greater excess of amines at N/P 8 was preferred for complex stability in protein-rich media. In serum stability assays, the siRNA inside N/P = 8 particles remained intact throughout 24 h of incubation, while free siRNA degraded progressively under the same conditions (Figure 4D; semiquantitative band comparison on the single archived gel). The selected formulation had a tight size distribution (polydispersity index 0.19–0.32; Figure 4E), a stable zeta potential (Figure 4F), and a stable particle size for 14 days at 4 °C (Figure 4G). Negatively stained transmission electron micrographs of the final N/P = 8 formulation revealed a spherical particle morphology, with dry-state dimensions expectedly smaller than the DLS hydrodynamic diameters (Figure 4H). This formulation, SM-102-based at N/P = 8 with 1.5% DMG-PEG2000, was used in all subsequent experiments.

### 3.5. LNP-Mediated JAG1 Knockdown Suppresses the Formation of a HemSC Network In Vitro

**Figure 5:**
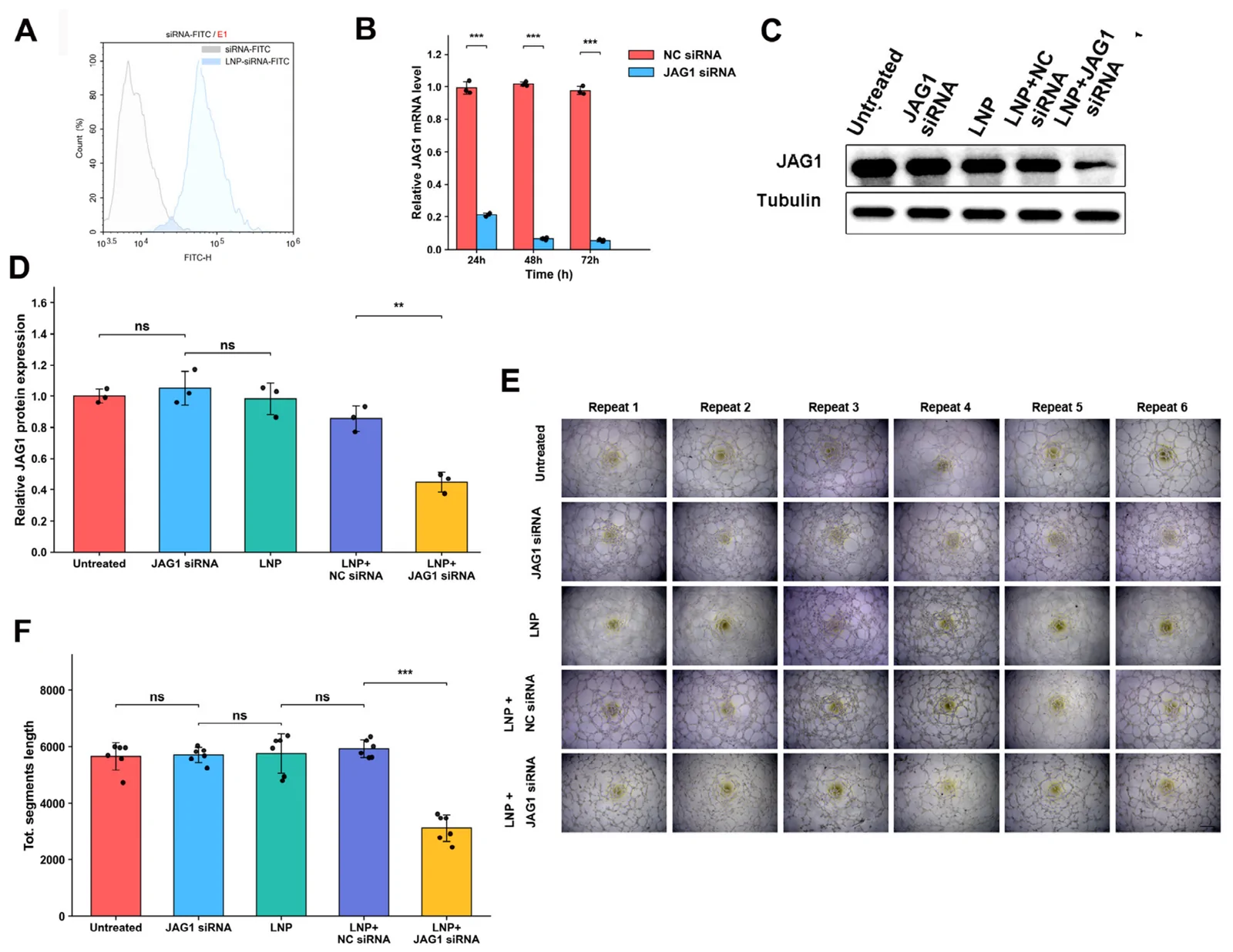
In vitro knockdown and functional validation. (**A**) Binding of FITC-labeled siRNA-LNPs to HemSCs, quantified by flow cytometry in the FITC channel, with free FITC-siRNA as the control. (**B**) qRT–PCR of JAG1 mRNA at 24, 48, and 72 h after LNP+JAG1 siRNA versus LNP+NC siRNA, expressed using the 2^−ΔΔCT^ method with 18S as an internal reference. (**C**,**D**) Western blot and densitometric quantification of JAG1 protein expression normalized to that of tubulin. (**E**,**F**) Representative images of HemSC network formation on Matrigel and quantification of total segment length using ImageJ. Scale bars, 100 µm. The data in (**B**–**D**) are presented as the mean ± SD of three independent experiments; (**E**,**F**) show six technical replicate wells per group. ** *p* < 0.01, *** *p* < 0.001 versus LNP+NC siRNA. ns, not significant.

Next, we asked what the particles do to cells. FITC-labeled siRNA-LNPs bound HemSCs efficiently (Figure 5A; quantified by flow cytometry in the FITC channel). qRT–PCR revealed that LNP+JAG1 siRNA, but not LNP+NC siRNA, decreased JAG1 mRNA levels at 24, 48, and 72 h after transfection (Figure 5B); two-way ANOVA confirmed a significant treatment × time interaction (F(2, 12) = 501.7, *p* = 2.7 × 10^−12^), with knockdown deepening from 78% at 24 h to 93% at 48 h and 95% at 72 h, while the negative control remained stable. Western blots revealed the same results at the protein level (Figure 5C,D; uncropped membranes in Figure S7A). Functionally, JAG1-silenced HemSCs formed clearly shorter networks on Matrigel, which was quantified as the total segment length (Figure 5E,F). CCK-8 assays under identical treatment conditions revealed no significant difference in viability among the four groups at the network endpoint (4–6 h: 93.2% of untreated; one-way ANOVA, *p* = 0.41) or at 24, 48, and 72 h (96.5%, 97.5%, and 97.3% of untreated; *p* = 0.66, 0.88, and 0.85, respectively; n = 3 independent experiments), indicating a network-specific effect rather than reduced cell viability. HemSC network formation on Matrigel therefore depends on JAG1 in this model. With respect to delivery, knockdown, and phenotype, the LNP carried the siRNA, JAG1 expression decreased at both the transcript and protein levels, and the network-forming phenotype decreased. These results justified the use of an in vivo model.

### 3.6. LNP-Mediated JAG1 Silencing Suppresses Hemangioma Growth In Vivo

We subsequently performed the silencing work in vivo. In a separate cohort, tumor-bearing mice received a single intratumoral injection of LNP+JAG1 siRNA at 5 mg/kg, with LNP+NC siRNA, LNP alone, and untreated mice as controls. The tumors were harvested 48 h later and allowed to clear. Densitometric quantification with JAG1 normalized to tubulin revealed that the LNP+JAG1 siRNA group expressed approximately half the JAG1 protein of the untreated control (~55%), whereas the JAG1 siRNA, LNP, and LNP+NC siRNA groups expressed JAG1 at levels comparable to or slightly above those of untreated tumors (Figure 6A,B; uncropped membranes in Figure S7B). The carrier, not naked siRNA, delivers the payload where it needs to go, and the in vivo knockdown matches the in vitro data.

The antitumor experiment lasted 35 days. The tumors in all four control groups grew steadily; however, those in the LNP+JAG1 siRNA group lagged behind those in the early group (Figure 6C). At the day 35 endpoint, the mean tumor volume was 67 ± 12 mm^3^ in the LNP+JAG1 siRNA group versus 280 ± 61 mm^3^ in the control group, 259 ± 49 mm^3^ in the JAG1 siRNA group, 260 ± 78 mm^3^ in the LNP group, and 248 ± 45 mm^3^ in the LNP+NC siRNA group, a reduction of approximately 75% (Figure 6D). The tumor weights were 55 ± 10 mg versus 280 ± 45 mg, 290 ± 42 mg, 300 ± 45 mg, and 267 ± 25 mg in the four control groups, approximately 80% lower (Figure 6E).

Body weight remained stable in every group (Figure 6F); thus, we observed no overt toxicity on the measures collected (body weight and clinical observation), and blood chemistry and organ histology remain to be evaluated. The microvessel density (MVD), which was calculated as the number of lumens containing red blood cells on H&E sections, decreased to 125 ± 22 vessels/mm^2^ (mean ± SEM) in the LNP+JAG1 siRNA group from 265 ± 80 (untreated), 263 ± 42 (JAG1 siRNA), 293 ± 45 (LNP), and 280 ± 33 (LNP+NC siRNA) vessels/mm^2^ (Figure 6G). New CD31 immunohistochemistry of the archived xenograft sections was qualitatively consistent with a reduced endothelial compartment in the LNP+JAG1 siRNA group (Figure 6I). Because only one archived section per group was available for the new staining, the CD31 findings are reported qualitatively: field-level measurements within a single section cannot separate between-section variability from treatment effects, so no inferential statistics were computed from them, and quantitative vascular inference remains anchored to the blinded, animal-level H&E-based microvessel density (Figure 6G). H&E staining confirmed the lesional vascular architecture of the hemangioma (Figure 6H), and GLUT1 immunostaining of the archived sections confirmed the GLUT1-positive phenotype of the lesion cells (Figure S8).

The amount of JAG1 protein decreased by approximately half, the microvessel density decreased by more than 50%, and the tumor volume and weight decreased by 75% to 80%, with no weight loss. Molecular knockdown, functional readout, and macroscopic outcomes are in agreement with one another. JAG1 is a real driver of hemangioma angiogenesis, and LNP-delivered siRNA against JAG1 was used in this model.

## 4. Discussion

IH is a vascular tumor whose growth phase involves two processes, angiogenesis and the cell cycle. By mining the GSE127487 transcriptome by differential expression analysis, weighted gene coexpression network analysis (WGCNA), and protein–protein interaction (PPI) network analysis, we identified JAG1, a principal endothelial Notch ligand, at the junction of both. In the PPI network constructed from the full DEG list, JAG1 is the single most connected node (degree rank 1 of 828) and anchors a Louvain cluster whose top GO terms are Notch signaling and its regulation, whereas the trait-correlated ME3 module is dominated by endothelial identity genes (CDH5, PECAM1, CD34, TEK, and ENG); JAG1 itself was uniformly and robustly upregulated across IH specimens. A Notch ligand that is associated with TOP2A, a canonical mitotic kinase among the genes most strongly induced in the lesion (logFC = 4.72; Figure 1A), suggests that JAG1 couples angiogenic Notch signaling to the proliferative machinery of IH rather than merely accompanying it.

JAG1 is a robust DEG but not a member of the ME3 module; curated-set GSEA within the corrected module revealed no significant enrichment of Notch, whereas sample-level ssGSEA revealed persistent Notch gene-set scores across all three clinical stages. The two methods measure different things, enrichment within module genes versus whole-transcriptome pathway activity per sample, so they need not agree. Nevertheless, the contrast suggests that Notch-related genes are not densely coexpressed inside the IH-correlated module, and cell type-specific work is needed. Our persistent Notch gene-set signature stands in apparent contrast to the individual-gene Notch switch reported to accompany involution [9]. Both findings suggest that the late role of Notch is maintenance: endothelial quiescence and vascular maturation. JAG1/Dll4 balance governs tip-versus-stalk fate decisions during sprouting [22,24], and endothelial JAG1 promotes maturation while antagonizing Dll4-driven branching [23]. Because downstream activity markers (cleaved Notch intracellular domain and HEY1) were not assessed, the mechanistic statements are limited to JAG1-silenced phenotypes; at the network level, the evidence of Notch is component-based (a JAG1-centered Notch signaling cluster in the DEG-level PPI) rather than activity-based.

The functional experiments revealed that JAG1 moved from the marker to the driver. Silencing it in HemSCs reduced network formation on Matrigel in vitro; silencing it in vivo reduced tumor growth and microvessel density without weight loss. These data extend the work of Boscolo and colleagues, who showed that Jagged1 governs the differentiation of hemSCs into pericytes and vascular smooth muscle [26], leading to endothelial behavior in the lesion itself. Which cells supply the JAG1 signal matters here: the knockdown series detects JAG1 mRNA and protein in the cultured HemSCs themselves, so the in vitro phenotype tests the cell-intrinsic (autocrine/juxtacrine) contribution of the ligand, whereas the in vivo phenotype after intratumoral delivery may also involve paracrine effects on host endothelium and other lesion cells; cell-type-resolved experiments are required to apportion these contributions. The antitumor effect tracks the delivery system precisely: naked JAG1 siRNA did nothing, LNP alone did nothing, LNP+NC siRNA did nothing, and only LNP+JAG1 siRNA worked. The numerically elevated H&E-based MVD in the two LNP-bearing control groups was not statistically significant; therefore, any carrier-associated vascular effect remains unestablished and deserves dedicated study. Candidate contributors—repeated intratumoral injection with local tissue trauma, a lipid-associated local inflammatory response to the SM-102-based carrier, the lipid composition itself, or chance variation in a small cohort—cannot be distinguished in the present design. If the carrier itself is mildly proangiogenic, that matters for how such formulations should be designed and dosed.

The formulation has practical strengths. Making blank particles first and adding siRNA afterwards means that the RNA never touches the organic solvent; loading is then efficient at a moderate N/P ratio of 8. SM-102 already has a clinical track record in validated mRNA-LNP formulations [28,51], and it gave our particles pH-dependent endosomal escape and robust serum nuclease protection for at least 24 h [52,53]. In terms of twice-daily oral propranolol and its systemic adverse effects [13,14], repeated local intratumoral dosing aimed at one chosen target is an attractive option for lesion-confined treatment. Our earlier rapamycin systems for hemangioma [30,31,32] and our siRNA platforms across multiple cancer types [33,34,35,37,39,40] made this step short rather than rapid.

Translation would sensibly keep the local route first. Superficial IH lesions are clinically amenable to intralesional therapy, and intralesional corticosteroid injection has a long-standing precedent in lesion-directed treatment [54]; thus, a locally injected siRNA-LNP would map onto an existing care pathway rather than require a new one. Systemic delivery, by contrast, would need active targeting to avoid uptake by the reticuloendothelial system and remains explicitly untested here.

How does JAG1 silencing compare with existing and emerging options? Propranolol promotes apoptosis in hemangioma endothelium through p53-BAX and mitochondrial pathways [10,11,55,56]; our approach involves activating Notch signaling. Different levels of the same vasculature. Emerging work on noncoding RNAs, such as miR-126, points to the layered regulation of IH proliferation and to possible biomarkers [57,58,59], and antiangiogenic siRNA-loaded nanoparticles have already proven themselves in other tumor contexts [60]. Endothelial JAG1 also supports solid tumor growth through proangiogenic and angiocrine functions [61], so the axis we target here is not unique to IH. It is a conserved angiogenic dependency that is targetable in principle, although its therapeutic value outside IH remains untested.

Placing these results alongside propranolol requires care because the present study included no direct head-to-head comparisons. Propranolol remains the reference standard for IH [3,13] and acts through systemic β-adrenergic blockade with twice-daily oral dosing [12,13], whereas JAG1-siRNA-LNP silences a local Notch ligand at the injected lesion; the two therefore differ in mechanism, route and exposure profile rather than competing on the same axis. In terms of mechanistic complementarity, combination rather than replacement is the rational long-term goal [18], but the formal additivity, dosing and safety of such combinations remain untested, as does performance against propranolol itself. Until such data exist, JAG1-siRNA-LNP should be read as a complementary, lesion-directed preclinical strategy rather than a substitute for standard treatment.

This study has several limitations that we want to put on the table. Bioinformatics relies on a single public microarray series with 18 IH and 5 control samples; thus, larger prospective cohorts should be used to confirm the hub structure. The series also provides no covariates beyond stage (only tissue, age, and propranolol status, with age collinear with stage); thus, residual confounding cannot be addressed by covariate adjustment. Cell types cannot be resolved from bulk tissue, and our functional work used HemSCs rather than hemangioma-derived endothelial cells; repeating the knockdown phenotype in HemECs with cell type-resolved approaches [7,8] would elucidate the underlying mechanism. GLUT1 immunostaining of the cultured HemSC isolate itself was not performed; thus, the GLUT1-positive identity of the cells rests on lesional immunohistochemistry together with CD133 selection purity rather than on direct staining of the cultured isolate. The xenograft model does not reproduce human involution kinetics, and the group sizes were modest. The off-target effects of the siRNA, the absence of a second JAG1-targeting siRNA or rescue construct, and the long-term safety, biodistribution, and immunogenicity of LNPs, including complement activation and anti-PEG responses that govern LNP pharmacokinetics [28,29], remain to be determined. Single-cell and spatial transcriptomics [62,63], JAG1 colocalization with endothelial and mural markers, more faithful IH models, and combination with propranolol are the obvious next steps. Systemic toxicity cannot be excluded from body weight alone; blood chemistry and organ histology are needed in future evaluations. Ki67/TUNEL proliferation and apoptosis staining was not performed on the xenograft sections, so a direct vascular effect cannot yet be separated from the smaller tumor mass; cell type-resolved, animal-level vessel quantification (quantitative CD31 immunohistochemistry across multiple sections per animal) and proliferation/apoptosis markers are needed. The flow-cytometric FITC readout (Figure 5A) shows the cell-associated signal without distinguishing surface-bound particles from internalized particles; confocal z-stacks with trypan blue quenching are the next steps. The formulation screen compared N/P ratios on physicochemical grounds; a head-to-head functional comparison across N/P ratios was not performed, and the working selection (N/P = 8) was validated functionally in the JAG1 knockdown series. The serum stability readout rests on a single archived gel scored semiquantitatively, and a fluorescence-based intact-siRNA assay on independent replicates would provide a properly quantitative endpoint.

## 5. Conclusions

In short, JAG1 is markedly and persistently upregulated in infantile hemangioma, sits at the hub of its transcriptomic network, and, when silenced by LNP-delivered siRNA, reduces the formation of the HemSC network on Matrigel in vitro and tumor growth with angiogenesis in vivo. In this preclinical model, the target is supported in principle, and the carrier performs. Locally delivered JAG1-directed RNAi remains a preclinical proposition for lesion-specific therapy for infantile hemangioma—pharmacokinetics, systemic exposure, repeated-dose safety, and clinically relevant delivery—and perhaps for other JAG1-dependent angiogenic diseases.

## Data Availability

The GSE127487 dataset analyzed in this study is publicly available from the NCBI Gene Expression Omnibus (https://www.ncbi.nlm.nih.gov/geo/, accessed on 25 August 2026). Other data supporting the findings of this study are available from the corresponding author upon reasonable request. Key processed analysis outputs (per-stage differential-expression tables and gene-set definitions) are provided in the revised Supplementary Material (Tables S1 and S2); the remaining analysis scripts and metadata are available from the corresponding author upon reasonable request.

## Supplementary Materials

The following supporting information can be downloaded at https://www.mdpi.com/article/10.3390/pharmaceutics18101322/s1. Figure S1: Gene set enrichment analysis of the IH transcriptome; Figure S2: Expression of the 20 hub genes from the ME3/DEG overlap in IH versus normal tissue; Figure S3: Heatmap of the endothelium-development Louvain cluster (Cluster 1); Figure S4: Heatmap of the Notch signaling cluster from the DEG-level PPI (JAG1-centered). Figure S5: Stage-annotated sample distribution. Figure S6: Corrected module GSEA curves. Figure S7: Uncropped full-membrane western blots. Full uncropped membrane scans for every western blot in the study were retained as archived. Figure S8: Representative GLUT1 immunohistochemistry results for each treatment group. GLUT1 immunohistochemistry of archived xenograft sections. Table S1: Gene sets used for ssGSEA (and module GSEA). Table S2: Stage-stratified differential expression (GSE127487).

## Abbreviations

The following abbreviations are used in this manuscript: IH, infantile hemangioma; LNP, lipid nanoparticle; siRNA, small interfering RNA; DEG, differentially expressed gene; WGCNA, weighted gene coexpression network analysis; PPI, protein–protein interaction; GO, Gene Ontology; KEGG, Kyoto Encyclopedia of Genes and Genomes; ORA, overrepresentation analysis; GSEA, gene set enrichment analysis; ssGSEA, single-sample GSEA; HemECs, hemangioma-derived endothelial cells; qRT–PCR, quantitative real-time PCR; MVD, microvessel density; HemSC, hemangioma stem cell; VEGF, vascular endothelial growth factor.
