# Supplementary material for: JAG1-siRNA-Lipid Nanoparticles Suppress Infantile Hemangioma Angiogenesis
Source: Pharmaceutics. 2026 Oct 19;18(10):1322. doi: 10.3390/pharmaceutics18101322 (PMC13708042; doi:10.3390/pharmaceutics18101322)
Supplement: Supplementary file 1 [file pharmaceutics-18-01322-s001.zip › pharmaceutics-4555939-supplementary.pdf]

Supplementary Materials

Figure S1. Gene set enrichment analysis of the IH transcriptome.

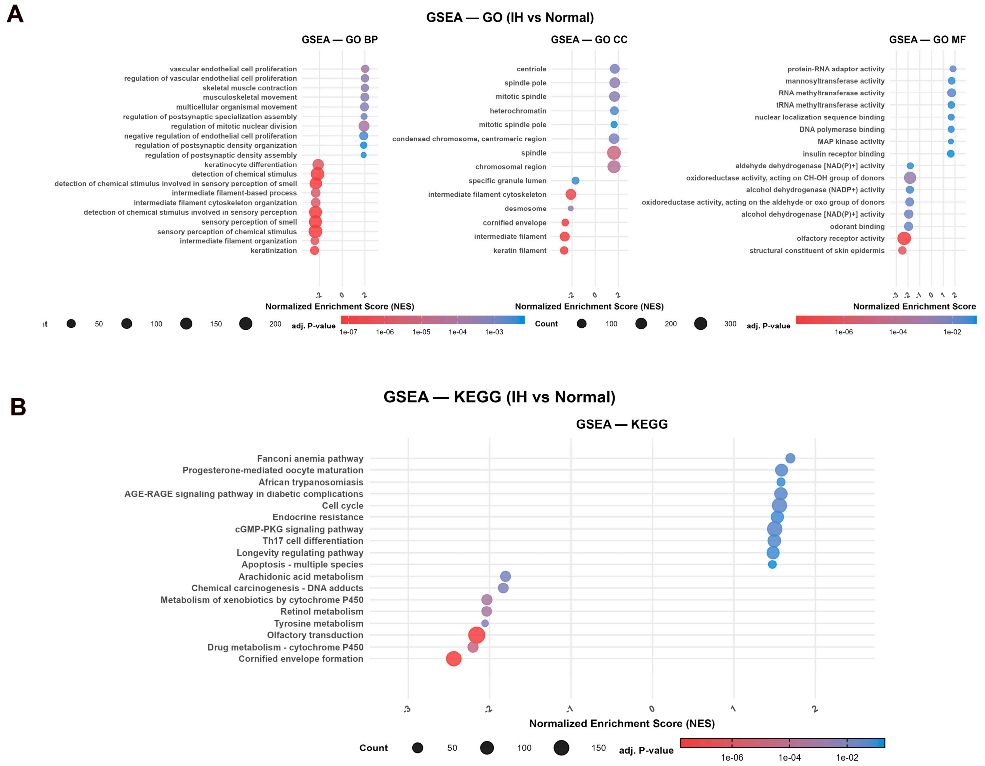

Figure S2. Expression of the 20 hub genes from the ME3/DEG overlap in IH versus normal tissue.

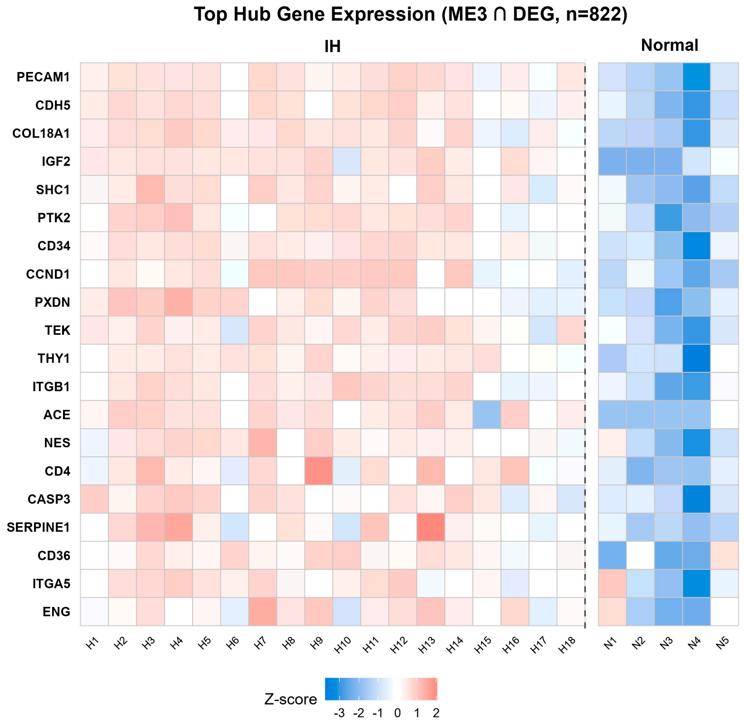

Figure S3. Heatmap of the endothelium-development Louvain cluster (Cluster 1).

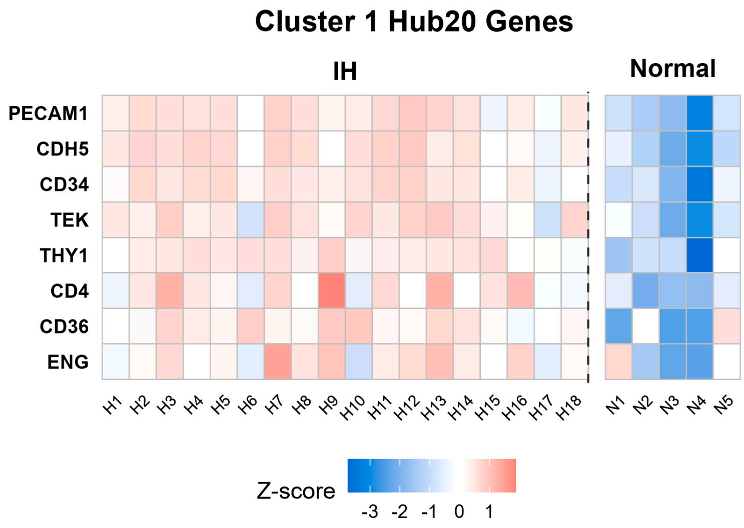

Figure S4. Heatmap of the Notch signaling cluster from the DEG-level PPI (JAG1-centered).

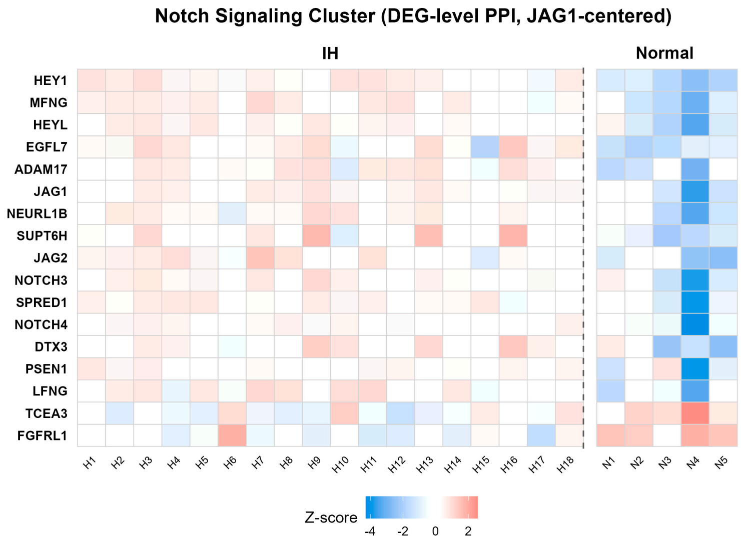

Figure S5. Stage-annotated sample distribution. (A) PCA of all 23 samples colored by group/stage; (B) JAG1 expression by stage.

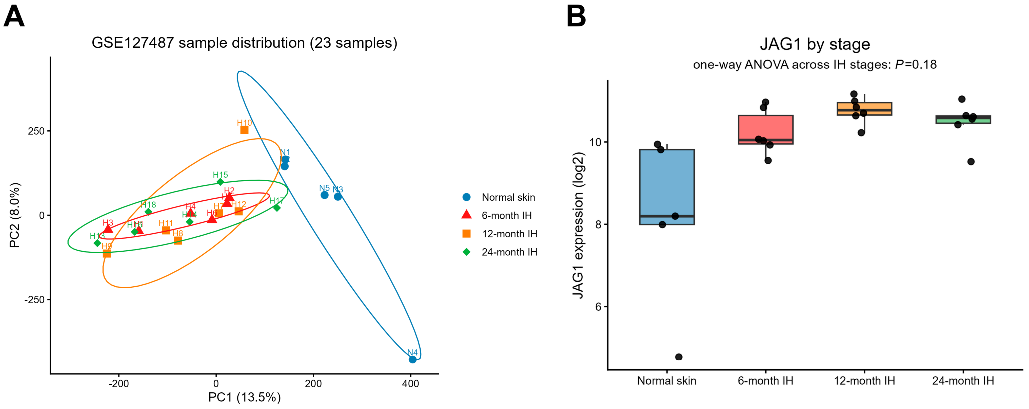

Figure S6. Corrected module GSEA curves.

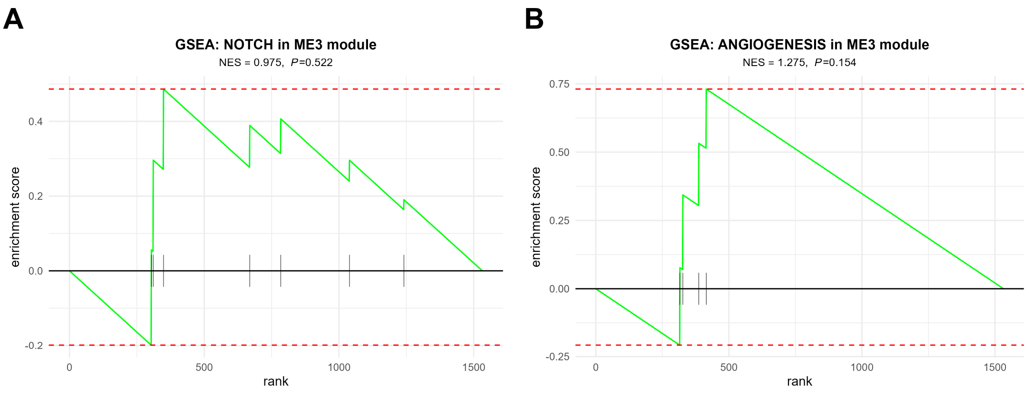

Figure S7. Uncropped full-membrane western blots. Full uncropped membrane scans for every western blot in the study were retained as archived; (A) In vitro HemSC blots (source of Figure 5C–D). (B) In vivo xenograft blots (source of Figure 6A–B). Bands were assigned by the expected molecular weight (JAG1 ≈134 kDa; α-tubulin ≈50 kDa); the molecular-weight marker run on every gel (Western Protein Marker I, Servicebio G2086) is documented in the experimental records.

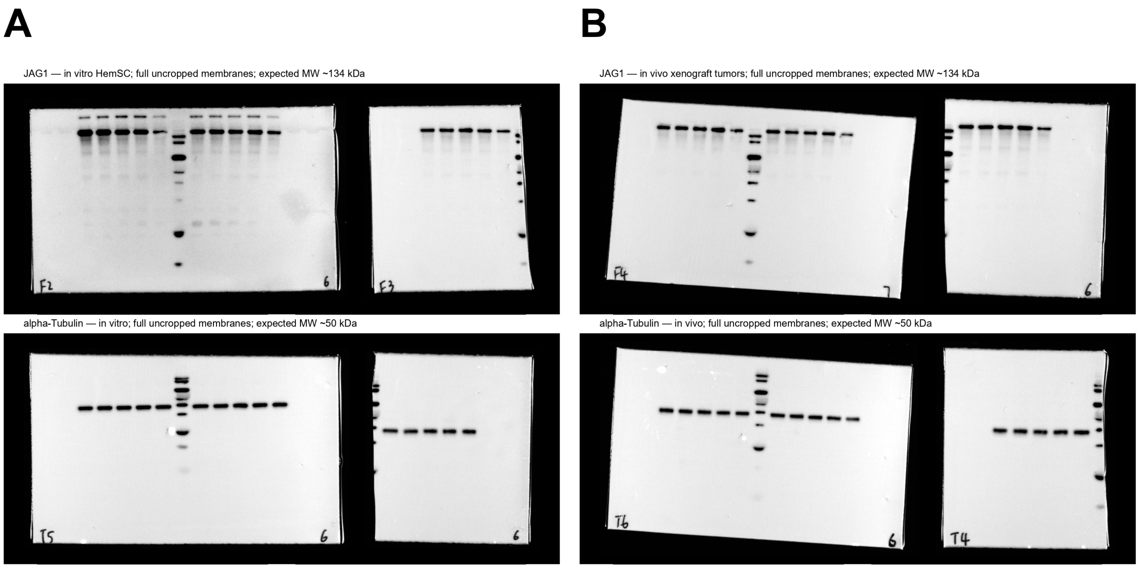

Figure S8. Representative GLUT1 immunohistochemistry results for each treatment group. GLUT1 immunohistochemistry of archived xenograft sections. Representative GLUT1 immunohistochemistry image, one representative field per treatment group (20× scans). GLUT1 immunostaining confirmed the GLUT1-positive phenotype of the lesion cells across the newly stained sections.

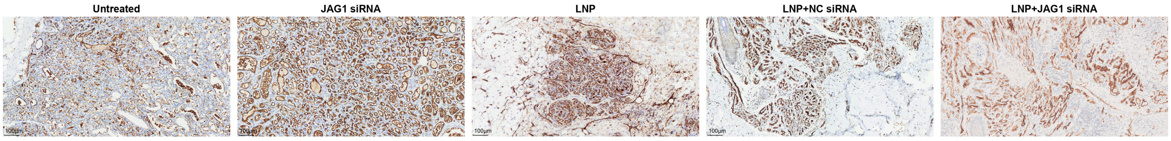

Table S1. Gene sets used for ssGSEA (and module GSEA).

Set	n	Genes
NOTCH	20	JAG1, JAG2, NOTCH1, NOTCH2, NOTCH3, NOTCH4, DLL1, DLL4, HEY1, HEYL, HES1, RBPJ, LFNG, MFNG, ADAM10, ADAM17, PSEN1, PSENEN, NCSTN, APH1A
PROLIFERATION	10	AURKA, AURKB, TOP2A, UBE2C, KIF20A, CDCA5, HJURP, CEP55, TACC3, MKI67
ANGIOGENESIS	14	ANGPT2, VEGFA, VEGFB, VEGFC, KDR, TEK, PLAU, ETS1, NOTCH4, JAG1, FYN, MMP9, MMP2, HIF1A
ECM	7	COL1A1, COL3A1, COL5A2, COL6A3, FN1, ITGA3, ITGB1
ME3 module	1531	Full list deposited with the revision data

Note: GSEA input: verified ME3 module genes ranked by IH-vs-normal log2FC (fgsea, 10,000 permutations, seed 42). Module results: NOTCH NES = 0.975, P = 0.52; angiogenesis NES = 1.275, P

= 0.15; ECM below minimum size (2 members); PROLIFERATION has no module members. ssGSEA across IH stages (fixed sets above): ECM ANOVA $P = 0.022$; proliferation $P = 0.076$; Notch $P = 0.52$; angiogenesis $P = 0.19$.

Table S2. Stage-stratified differential expression (GSE127487).

Comparison	UP	DOWN	JAG1 log2FC	JAG1 P	JAG1 BH-adjusted P
6-month vs normal	331	39	2.084	0.033	0.258
12-month vs normal	212	37	2.616	0.0099	0.206
24-month vs normal	73	23	2.32	0.02	0.305
Pooled IH vs normal	1753	213	2.34	1.4×10^{-4}	0.006

Note: limma, each stage (n = 6) vs normal skin (n = 5); |log2FC| > 1 and BH-adjusted $P < 0.05$. JAG1 statistics are shown per stage and are pooled (18 vs 5). Shared upregulated genes across all three stages: 35; shared downregulated genes: 0. The per-stage adjusted P values did not significantly differ for individual genes at n = 6 (power limitation); the pooled comparison and consistent direction across stages support sustained JAG1 expression.
